# Investigating Microtemporal Processes Underlying Health Behavior Adoption and Maintenance: Protocol for an Intensive Longitudinal Observational Study

**DOI:** 10.2196/36666

**Published:** 2022-07-14

**Authors:** Shirlene Wang, Stephen Intille, Aditya Ponnada, Bridgette Do, Alexander Rothman, Genevieve Dunton

**Affiliations:** 1 Department of Population and Public Health Sciences Keck School of Medicine University of Southern California Los Angeles, CA United States; 2 Khoury College of Computer Sciences Northeastern University Boston, MA United States; 3 Bouvé College of Health Sciences Northeastern University Boston, MA United States; 4 Department of Psychology University of Minnesota Minneapolis, MN United States

**Keywords:** emerging adulthood, behavior change, longitudinal data collection, ecological momentary assessment, sensing, theory, young adult, weight gain, EMA, chronic disease, physical activity

## Abstract

**Background:**

Young adulthood (ages 18-29 years) is marked by substantial weight gain, leading to increased lifetime risks of chronic diseases. Engaging in sufficient levels of physical activity and sleep, and limiting sedentary time are important contributors to the prevention of weight gain. Dual-process models of decision-making and behavior that delineate reflective (ie, deliberative, slow) and reactive (ie, automatic, fast) processes shed light on different mechanisms underlying the adoption versus maintenance of these energy-balance behaviors. However, reflective and reactive processes may unfold at different time scales and vary across people.

**Objective:**

This paper describes the study design, recruitment, and data collection procedures for the Temporal Influences on Movement and Exercise (TIME) study, a 12-month intensive longitudinal data collection study to examine real-time microtemporal influences underlying the adoption and maintenance of physical activity, sedentary behavior, and sleep.

**Methods:**

Intermittent ecological momentary assessment (eg, intentions, self-control) and continuous, sensor-based passive monitoring (eg, location, phone/app use, activity levels) occur using smartwatches and smartphones. Data analyses will combine idiographic (person-specific, data-driven) and nomothetic (generalizable, theory-driven) approaches to build models that may predict within-subject variation in the likelihood of behavior “episodes” (eg, ≥10 minutes of physical activity, ≥120 minutes of sedentary time, ≥7 hours sleep) and “lapses” (ie, not attaining recommended levels for ≥7 days) as a function of reflective and reactive factors.

**Results:**

The study recruited young adults across the United States (N=246). Rolling recruitment began in March 2020 and ended August 2021. Data collection will continue until August 2022.

**Conclusions:**

Results from the TIME study will be used to build more predictive health behavior theories, and inform personalized behavior interventions to reduce obesity and improve public health.

**International Registered Report Identifier (IRRID):**

DERR1-10.2196/36666

## Introduction

Engaging in sufficient levels of physical activity [1] and sleep [2], and limiting sedentary time [3] are important contributors to the prevention of weight gain and decreased lifetime risks of cancer, diabetes, cardiovascular disease, and mortality [4-7]. However, engagement in these healthy behaviors steeply declines during young adulthood (ages 18-29 years) [8-10]. Existing interventions designed to promote physical activity, reduce sedentary time, and support sufficient sleep among young adults typically focus on the *adoption* of these behaviors. Yet, often, when these interventions are successful, new patterns of behavior are not *maintained* and regress back to baseline levels [11]. Temporary disengagements are frequent among individuals attempting to maintain healthy behaviors, but little is known about how to help individuals avoid or manage these disruptions [12].

The first generation of health behavior theories provide limited guidance regarding factors underlying the transition from initiating to maintaining a pattern of behavior [13-16]. More recently, dual-process models of decision-making and behavior have offered explanations for different mechanisms underlying adoption versus maintenance [17-19]. Reflective processes, which are slow and deliberative (eg, deliberating, evaluating one’s efficacy, exerting self-control) [20-23], may be engaged to a greater extent when adopting a behavior, whereas reactive processes, which are fast and automatic (eg, contextual cues, habits) [24,25], may be more involved in behavior maintenance. Thus, understanding the independent and interactive effects of reflective and reactive factors may afford more precise predictions of behavior adoption and maintenance [26,27].

The dynamic and idiographic properties that characterize reflective and reactive processes, which may change dynamically, within a day, and differently across individuals [28,29], are not well captured using static, cross-sectional, laboratory-based, or retrospective research methods [30]. The application of methods and tools for collecting and analyzing intensive longitudinal data (ILD) may enable better research on factors influencing reflective and reactive processes, and thus support new theory and intervention development. ILD are collected from real-world settings in temporally dense micro time scales (eg, seconds, minutes, hours). Improved miniaturization, capability, affordability, and pervasiveness of mobile and wearable devices in recent years have enabled the capture of ILD.

In the Temporal Influences of Movement and Exercise (TIME) study, we are using real-time mobile technologies (consumer-grade smartphones and smartwatches) to collect ILD to examine differences in the microtemporal processes underlying the adoption and maintenance of physical activity, low sedentary time, and sufficient sleep duration among young adults. We aim to predict within-subject variation in the likelihood of behavior “episodes” (eg, ≥10 minutes of physical activity, ≥120 minutes sedentary time, ≥7 hours sleep) and “lapses” (ie, not attaining recommended levels for ≥7 days) as a function of reflective and reactive factors. Overall, this study seeks to yield new insights into the behaviors, states, and contexts that influence health behavior and decision-making, and to build better predictive models that can be used to drive personalized interventions targeting a wide variety of health behaviors that can be implemented in real time. In this paper, we describe the TIME study protocol.

## Methods

### Design Overview

**Figure 1:**
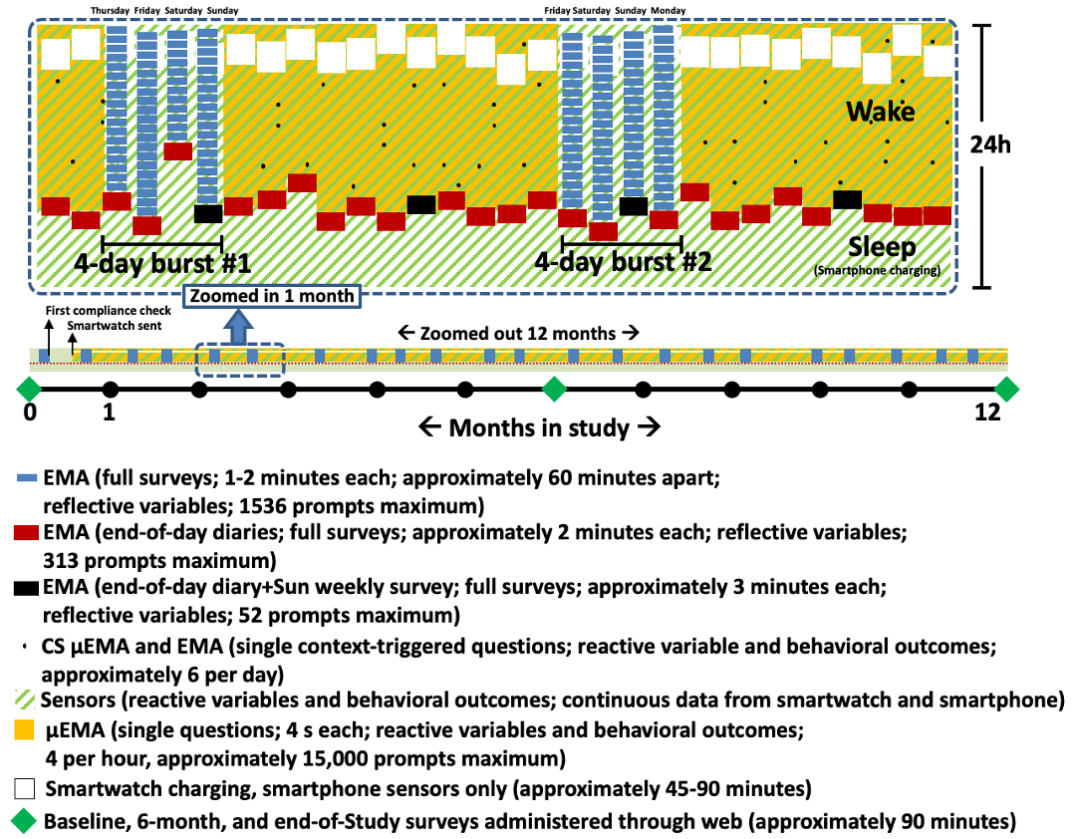
Temporal Influences of Movement and Exercise (TIME) study protocol with nested ecological momentary assessment (EMA) bursts and micro-EMA (μEMA) on nonburst days. ea: each; CS: context-sensitive.

The TIME study uses a prospective within-subject case-crossover observational design that collects ILD using smartphone and smartwatch technology worn continuously across a 12-month period in a sample of socioeconomically and racially/ethnically diverse young adults. In case-crossover designs, a subject serves as their own control to assess the within-subject effects of time-varying predictors and moderators on a repeatedly measured dependent variable. With this longitudinal design, the phase of behavior change (adoption vs maintenance) will vary between people and within people (who change over time). We are deploying a combination of continuous passive (sensor-based) and intermittent active (self-reported ecological momentary assessment [EMA]) monitoring methods (see Figure 1).

Across the 12-month study period, physical activity, sedentary behavior, and sleep outcomes are captured continuously using a smartwatch activity sensor. Time-varying reactive predictor variables (eg, location, phone/app use, time of day/week) are captured continuously using smartphone sensors and usage logging. To limit participant burden, time-varying reflective predictor variables (eg, self-control, demands, deliberation) are captured intermittently using self-report EMA (sets of questions administered together) with two types of prompting schedules (varying in prompting density): “measurement bursts” and “end-of-day (EOD) surveys.” Measurement bursts (lasting 4 days each) occur once every 2 weeks and EOD surveys occur each night. Additionally, data from context-sensitive (CS)-EMA prompting will be used to verify accuracy of location via passive sensor (GPS) data. During the nonmeasurement burst periods, participants are prompted on the smartwatch to gather additional data on reflective and reactive variables and behaviors, and are asked CS questions on physical activity and sedentary behavior (via raw accelerometer data) using micro-EMA (μEMA) [31]. Details of each component are described below.

### Participants

The study recruited young adults across the United States (N=246). Inclusion and exclusion criteria were assessed by self-report during the screening process. Inclusion criteria for the study were: (1) 18-29 years old living in the United States, (2) intention to engage in recommended levels of moderate-to-vigorous physical activity (MVPA) (≥150 minutes/week moderate or ≥75 minutes/week vigorous intensity) within the next 12 months, (3) use an Android-based smartphone as their only primary personal mobile device with no intention to switch to a non-Android phone, (4) able to speak and read English, and (5) plan to reside in a home with Wi-Fi connectivity during the study period. Exclusion criteria were (1) physical or cognitive disabilities that prevent participation; (2) health issues that limit physical activity; (3) any diagnosed sleep disorders; (4) unable to wear a smartwatch or answer EMA surveys at home, work, school, or another location where a substantial amount of time is spent; (5) spends more than 3 hours/day on a typical weekday or weekend day driving; (6) owns an Android phone version 6.0 (or older), or if the app will not function on the phone due to other technical issues; (7) currently owns and wears a smartwatch; (8) uses a pay-as-you-go data plan or data plan with less than 2 gigabytes of data per month; or (9) currently pregnant. Participants were recruited regardless of baseline activity level.

### Ethics Approval

The study was approved by the Institutional Review Board at the University of Southern California (USC; HS-18-00605). The study was performed in accordance with the ethical standards as laid down in the 1964 Declaration of Helsinki and its later amendments. All participants provided informed consent to have their deidentified data published in journals.

### Recruitment, Screening, Consent, and Orientation

Due to health and safety concerns arising from the COVID-19 pandemic, all study procedures were conducted remotely. To recruit socioeconomically and racially/ethnically diverse young adults, we used a variety of recruitment methods, which broadly sampled young adults across the United States. Recruitment strategies included the following: (1) sending emails to individuals enrolled in a USC longitudinal cohort study of young adults [32]; (2) referrals from existing participants (word of mouth); and (3) contacting participants identified using ResearchMatch, a national health volunteer registry [33]. Potential participants filled out an online interest form to screen eligibility. For eligible and interested potential participants, a videoconference orientation and consent session over Zoom was then scheduled. This session involved reviewing all parts of the study, obtaining informed consent, and downloading our custom TIME study smartphone app onto the participant’s smartphone (N=332). During the orientation session, participants received instructions on how to use the study app to complete EMA surveys. During the following week, individuals participated in their first 4-day EMA measurement burst period (further described below), during which the TIME app triggers surveys once per hour during the participant’s waking hours. Participants who successfully completed at least 8 surveys per day during this first EMA measurement burst period were fully enrolled in the study (N=246). If compliance was below 8 surveys/day for the first measurement burst period, participants were unenrolled from the study. Participants who were fully enrolled received a smartwatch by mail within 1 week and were scheduled for a second orientation session for smartwatch setup and training.

### Study App

EMA data are collected using our custom TIME app developed for Android smartwatches and smartphones. The app is downloaded directly to a participant’s personal Android phone from the Google Play Store but is only available to authorized study participants. Once the participant receives the smartwatch by mail, the TIME app is downloaded to the watch paired with the smartphone.

### Smartwatch

Participants are loaned a Fossil Sport Gen 4 or Gen 5 smartwatch. Participants are asked to wear the smartwatch on one wrist of their choice/comfort consistently and continuously over the study period, except for 1 hour per day when it should be charged by setting it on a provided charger. They are asked to develop a routine in which the smartwatch is fully charged every day, ideally at a consistent place and time such as during daily personal hygiene (eg, showering, bathing). Participants can use the smartwatch as they see fit throughout the study (eg, to get notifications from phone apps) if that use does not interfere with the TIME app’s functionality. Participants were allowed to install health and fitness apps on their personal smartphones, but we asked that participants refrain from installing these apps onto the smartwatch to preserve the battery life of the watch. These apps use motion data and the heart rate sensor, which cause quicker battery drain that would increase perceived study burden by having to charge the watch more than once a day. However, 22% of participants reported installing health and fitness apps on the watch. We will be able to use data on smartwatch app installation as covariates in our analyses.

### Data Collection Procedures

#### Ecological Momentary Assessment

##### Overview

**Figure 2:**
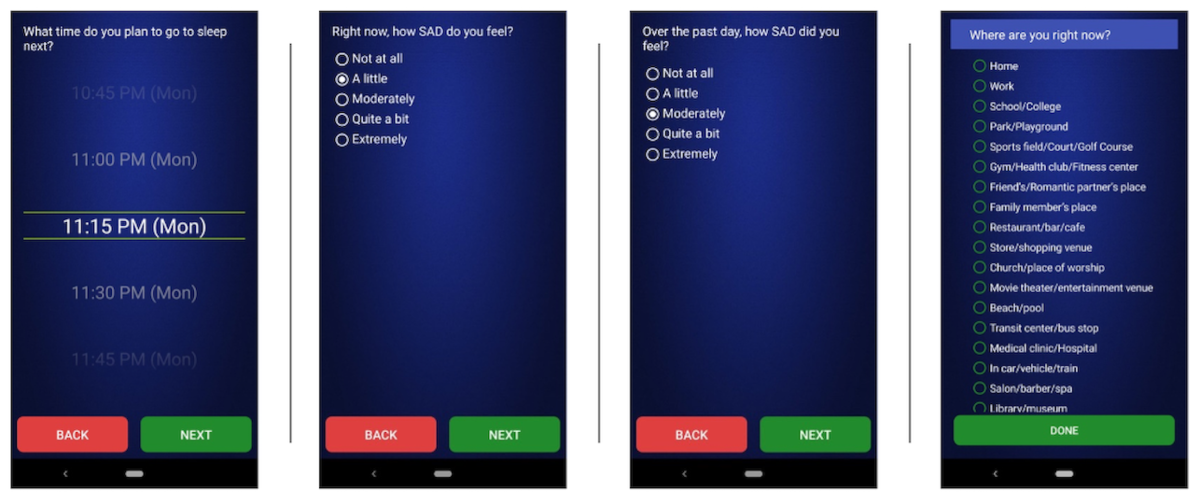
TIME app's ecological momentary assessment (EMA) interface, shown on a Google Pixel 3 phone. From left to right: example sleep time EMA measuring prospective sleep time, burst EMA question about momentary sadness, end-of-day EMA question about daily sadness, and context-sensitive location survey question assessing current location.

Participants complete EMA surveys on the smartphone and μEMA questions on the smartwatch throughout the study during waking hours. The smartphone uses push notifications to prompt participants to complete EMA question sets with back-to-back multiple-choice questions; question sets require ~1-2 minutes to complete. If a response is not provided, up to two reprompts will be emitted at 5-minute intervals; when the second reprompt answer time expires, the EMA survey becomes inaccessible and is recorded as missed. Once a smartphone survey is started, it must be completed within 10 minutes. If a smartphone or smartwatch prompt occurs during an incompatible activity (eg, driving, sleep), participants are instructed to ignore it. The smartwatch prompts single μEMA questions. A μEMA question can be answered in ~3 seconds with a glance and tap. Each μEMA question on the smartwatch must be answered within 20 seconds; there are no reprompts. EMA data are captured intermittently using sampling schedules varying in prompting density as described below: (1) sleep-wake time, (2) measurement bursts, (3) EOD surveys, and (4) sensor-informed CS prompting. Examples of these questions are shown in Figure 2.

##### Sleep-Wake EMA

EMA prompting takes place only during waking hours to prevent sleep disruption; however, unlike most prior work using EMA, waking hours are adapted dynamically to match each participant’s daily schedule. This outcome is achieved by asking participants to report their anticipated (ie, prospective) and actual (ie, retrospective) sleep/wake times each day. Sleep and wake times are assessed using short EMA surveys that are typically appended to other prompted surveys. During the EMA measurement burst days, the prospective sleep-wake questions are included in the first prompt of the day. Prospective sleep time questions are reassessed after 10 hours to capture potential changes in a participant’s schedule throughout the day. The retrospective sleep-wake questions are only prompted once a day. On nonburst days, the sleep-wake EMA surveys are prompted on the smartphone without additional EMA questions. When the sleep and wake times are changed by the participants, the TIME app automatically updates the EMA prompting schedules.

##### EMA Measurement Bursts

Each EMA measurement burst consists of signal-contingent (ie, randomly prompted) question sets triggered multiple times per day, approximately once every hour during waking hours across 4 consecutive days. Within 1 hour, the prompting is restricted to between the 10th and 50th minute to ensure that two prompts from consecutive hours do not occur too close to each other. EMA measurement bursts last 4 days each and measurement bursts occur every 2 weeks, resulting in up to 26 bursts during the study period (104 total days). During the measurement burst periods, participants continue to wear the smartwatch but do not receive any μEMAs. EMA measurement bursts occur on randomly scheduled blocks of days, with at least 7 days in between each burst and guaranteeing two weekends and two weekdays within each burst. One day before the burst is set to begin, the TIME app notifies the participants about their upcoming burst period via a phone notification that gives participants a chance to snooze (ie, delay) the beginning of the burst period by 2 days. Participants can snooze each burst period only once.

To promote compliance, once an EMA question set is completed, the app displays a lighthearted “thank you” message. There are 250 different EMA thank you messages, and therefore they rarely repeat, providing novelty after each question set. In addition, 20% of the EMA burst surveys include a validation question; these questions are designed to be entertaining and provide novelty (rarely repeating), but with unambiguous answers so that they can be used to determine whether participants are paying attention to EMA questions and answering thoughtfully.

The hourly sampling schedule used in the study was piloted with 45 participants for 1 month (two burst periods) before starting the data collection described in this protocol. Feasibility of the schedule was demonstrated with compliance rates of 77.0% (SD 16.7, range 41.5%-100%) for burst 1 and 78.9% (SD 16.1, range 35.8%-100%) for burst 2, which are similar to rates found in other EMA studies with less frequent prompting schedules [34]. For this study, compliance is defined as the number of completed surveys divided by the number of prompted surveys.

##### End-of-Day EMA

Across the 12 months, an EMA question set is prompted on the smartphone at the end of each day asking participants to summarize experiences occurring that day and their plans for the next day. Participants respond to EOD EMA prompts during both burst and nonburst study periods. EOD EMA prompts are delivered 2 hours before a participant’s anticipated sleep time. The question set remains accessible for 2 hours (up to the sleep time) or until it is answered via a persistent notification that can be clicked to access the EOD EMA survey. All EOD question sets ask about an individual’s anticipated sleep time that same day and anticipated wake-up time the next day. If the sleep time is extended more than 1 hour past the current time, the participant may receive more EMA measurement burst prompts after the completion of the EOD EMA question.

Once a week, on Sundays, the EOD EMA question sets include 28 additional unique questions asking about experiences over the past week, goals/intentions to engage in health behaviors in the upcoming week, and reactions to the COVID-19 pandemic.

##### Context-Sensitive EMA

On non-EMA measurement burst days, the smartphone also triggers sensor-informed CS-EMA surveys to gather data about the types of places where a participant is spending time. These surveys are prompted based on recorded information about a participant’s current and prior locations, measured using the phone’s location-sensing system. At the end of each day, a density-based clustering algorithm clusters that day’s location measurements [35,36]. When clusters are found, they are inserted into a master cluster list for the participant. If the app detects that the participant has spent at least 5 minutes in a previously identified cluster, if the type of that location is not known with high reliability (based on prior CS-EMA surveys for location) and if the participant has not been asked to label the location within the last 2 hours, the participant will be prompted to describe the type of the current location (ie, “Where are you right now?”). This single question includes 21 types of common locations (eg, home, work, park/playground/trail, church/place of worship, in car/vehicle/train). Once a location cluster has been reliably identified, the app no longer triggers a CS-EMA prompt when that location is reencountered, unless 60 days have elapsed since the last label for the cluster was obtained, in which case the participant will be asked to reconfirm the location type. Participants self-report their locations to attach more meaningful labels to commonly visited locations than can be obtained from a map application programming interface (API). As location data represent a key reactive factor being tested in the analyses, the precision of the label justifies the additional user burden.

##### Micro-EMAs

**Figure 3:**
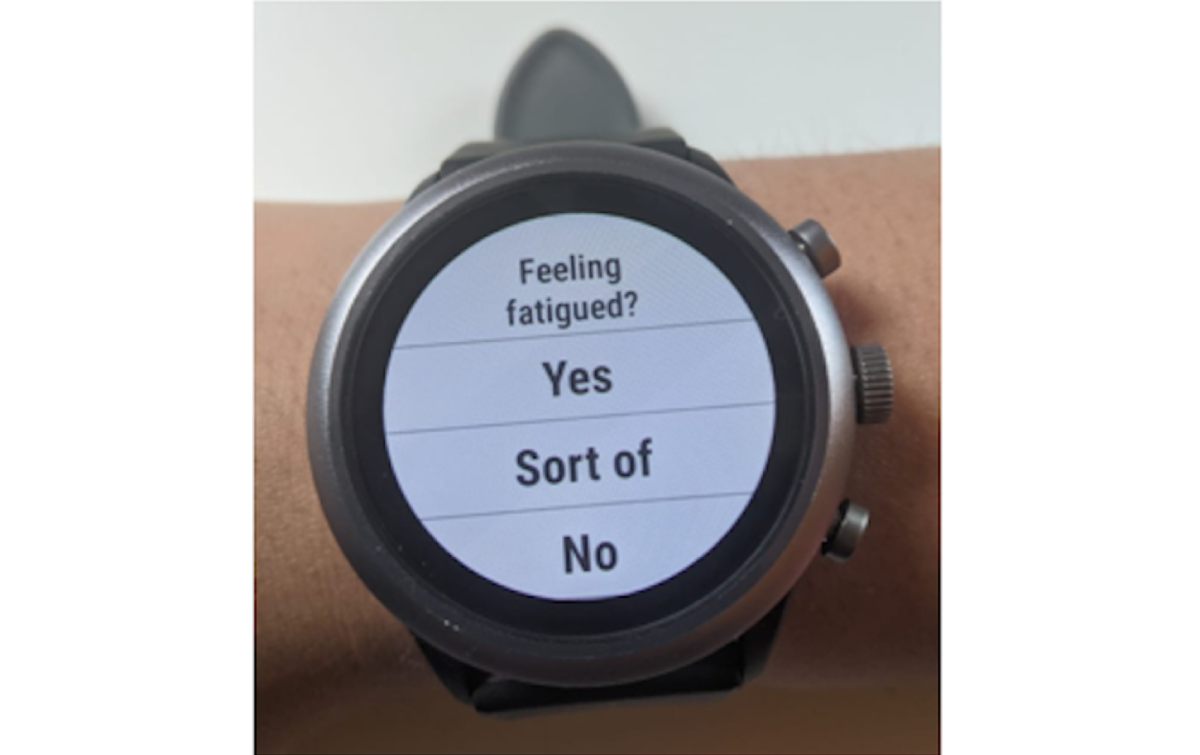
Example microinteraction ecological momentary assessment question on the smartwatch.

Outside of EMA measurement burst periods (on 261 days during the 12-month study), participants are prompted with μEMAs (also known as microinteraction EMAs or micro-EMAs) [31] throughout the waking day. Each μEMA prompt includes one single question that can be read with a glance and answered with a quick, single tap (Figure 3). μEMA questions have simple categorical/ordinal answer options (eg, “yes/sort of/no”) and are designed to be cognitively simple to answer (eg, Feeling stressed? Yes, Sort of, No). If the watch detects 10 minutes of continuous physical activity or 60 minutes of continuous sedentary behavior, sensor-triggered μEMA questions may be asked (eg, “physically active [x] min ago?” where “[x]” is the time difference between the prompt time and the middle of the window when the activity was detected). Additional details on the μEMA protocol and related study goals are described elsewhere [37].

##### Self-Report Online Electronic Questionnaires

Sociodemographic variables, mental health characteristics, health status, health behaviors, and other covariates are assessed at three time points (baseline, 6 months, 12 months) using online electronic questionnaires completed remotely on a computer, tablet, or smartphone. Questionnaire constructs include: general health [38], self-reported physical activity [39], usual lifestyle physical activities [40], team sports/activity classes [41], sedentary behavior [42], sleep problems [43], sleep disorders [44], diet [45], eating disorders [46], alcohol and substance use [47], acculturation [48], and demographics [38]. Participants receive an electronic link to the online questionnaire on REDCap (Research Electronic Data Capture) by email and are asked to complete the questionnaire within 7 days or before the end of the first burst period. Questionnaires take ~45-60 minutes to complete, and participants can stop midsurvey and return to the survey later to complete it.

At either the end of the 12-month period or when the participant is withdrawn or removed from the study, participants receive an email link to complete an additional 5-minute online end-of-study questionnaire about the acceptability of procedures and usability of the study app and smartwatch.

##### Exit Interview

At the end of the 12-month study period, participants complete a 30-45–minute interview on Zoom with study staff. Participants answer questions about the acceptability of study procedures, provide context to how they used their devices, and indicate how they interpreted and answered the study survey questions. This interview is recorded for transcription in subsequent analysis.

### Measures

#### Overview

We will collect data on reflective processes (eg, self-control, attention, procrastination, deliberation [“trying to decide”], and intention) using intermittent self-report through EMA. Some reactive factors (eg, lack of deliberation [“not thought about it”], habit [“doing usual routine”], and affective motivation [“feel like doing it”]) will also be captured through EMA. Continuous, sensor-based passive monitoring of reactive factors (eg, location and screen/app use) will also occur using smartwatches and smartphones.

#### EMA Items

All EMA questions are presented in Multimedia Appendix 1. Both EMA bursts and EOD EMA assess the following *global* constructs at every prompt (ie, occurring 100% of the time) using items taken directly or modified from established measures: affective and feeling states, stress, attention, self-control, productivity, and habit. During EMA bursts, items start with “Right now...” to capture momentary reports. For the EOD EMA, items start with “Over the last day...” to capture daily summaries. Two additional EMA burst items assess health behaviors and social contexts that cannot be detected from sensors: “Over the past hour, I did the following things (choose all that apply),” and “In the past hour, I was with (in person and/or virtual).”

To reduce the question set length, only one of four possible *behavior-specific* construct modules (ie, physical activity, sleep, sedentary behavior, eating) is included in each EMA burst and EOD EMA question set; the module is randomly selected (see proportions in Multimedia Appendix 1).

#### Passive Monitoring

Reactive factors that may influence physical activity, sedentary time, and sleep are acquired continuously via passive sensing using the participants’ smartwatch and smartphone sensors [49-51]. For any phone that permits it, each minute, the app collects light luminosity (measured in lux), ambient pressure (measured in hectopascal units), ambient relative humidity (measured as percentage), and temperature (measured in degrees Celsius). Once a day, using the UsageStats API in Android, the app captures the amount of time spent by the participant using different apps installed on the phone. Similarly, once an hour, the app saves the number of times different apps were opened (moved to the foreground) and closed (moved to the background). In addition, the app stores time-stamped data about phone use, such as phone unlocks, screen usage time, battery percentages, phone and watch charging events, and the notification frequency from other apps installed on the phone. The smartphone estimates the longitude and latitude of the participant each minute using the smartphone’s location system, except for when the phone is turned off.

All participants in the study were informed about the type of passive data collected and consented to the procedures. Any identifiable data collected using the smartphone’s location system are encrypted at the time of collection and only used in an identifiable way by the research team.

#### Motion Data

Motion data are collected using raw acceleration data processing, phone activity levels, and estimated step counts

Triaxial raw acceleration along the X, Y, and Z axes on both the smartphone and smartwatch is measured at a sampling rate of ~50 Hz using the embedded accelerometers. Smartwatch data are collected in the range of ±8 g; smartphone data sensitivity is based on the specific phone. The acceleration data are collected continuously except when the watch and/or phone are turned off. The area under the curve (AUC) of the summed 3-axis high-pass accelerometer signal is computed for 10-second epochs to provide a crude motion summary in real time on both the phone and the watch [34]. On the watch, this AUC value is used for sensor-triggered μEMA questions on physical activity and sedentary behavior.

The movement state of the smartphone (ie, the phone activity level) is captured each minute using Android’s activity recognition API. Using the phone’s motion sensors, an algorithm estimates if the user is “in vehicle,” “on bicycle,” “on foot,” “running,” “tilting,” “still,” “tilted,” and “walking.” The labels are not mutually exclusive, and thus the algorithm can report that the smartphone is “in vehicle” and “still” at the same time.

Once an hour, the number of steps recorded on the smartphone is collected using Android’s built-in step counter. This built-in counter uses the inertial sensors (accelerometer and gyroscope) to estimate the step count when the phone is not turned off.

### Data Processing Procedures

#### Motion Summary

Motion Independent Movement Summary (MIMS) units are computed using the raw acceleration data from the smartwatch after data collection. The watches reliably store raw accelerometer data at ~50 Hz. MIMS units are a device-independent summary of overall motion. The MIMS-unit algorithm is designed to allow for cross-monitor motion comparisons between research-grade devices such as actigraphs and consumer-grade devices such as the Fossil smartwatches used in this study [52]. MIMS units are computed with 1-second epochs, but can then be aggregated (eg, minute, hour, or day level). The SWaN (Sleep Wake and Nonwear) algorithm used in the study to summarize the raw accelerometer data has previously been used to summarize population-wide wrist-worn movement metrics in the NHANES data set [53]. Wrist-worn activity measurement may overestimate activity in response to large amounts of gesturing and underestimate activity for some activities such as cycling.

#### Smartwatch Sleep, Wear, and Sensor Nonwear

Sleep, wear, and sensor nonwear estimation is also computed after data collection using raw accelerometer data using the SWaN algorithm. SWaN classifies each 30-second window of the raw data into sleep, wear, and nonwear classes, each with some degree of certainty.

#### Smartwatch Activity Type

Finally, postprocessing using the watch accelerometer signal is used to estimate activity intensity (light, moderate, vigorous), posture (eg, sitting, standing, lying), and specific activities (eg, sitting and writing, walking, running). The algorithm classifies these activities for each 12.8-second window of raw data in the post data collection stages [54]. Estimates of MVPA will be developed using the passively collected smartwatch accelerometer data and these postprocessing algorithms.

#### Behavior Episode Categorization

For physical activity, labels will be assigned for each 1-minute interval, and any bout of MVPA ≥10 minutes will be considered a behavior episode based on the minimum recommended bout length for health benefits. Bouts of ≥120 minutes of sedentary time will be considered a behavior episode based on conferred health risks that start to emerge at this duration of prolonged sitting. Any period with ≥7 hours of sleep will be considered a behavior episode meeting the sleep guidelines for young adults.

#### Phase of Behavior Change Classification

Adoption versus maintenance phase will be a binary, time-varying variable assigned to each day in the study (starting on day 22) based on whether a participant has attained recommended levels of behavior/levels with conferred health benefits for ≥3 past weeks based on movement data collected from the smartwatch: adoption (not attained) or maintenance (attained). Alternative lengths of time (eg, 4 weeks, 6 weeks, 12 weeks) will be explored through sensitivity analyses. Thus, individuals can transition from adoption to maintenance or from maintenance to adoption throughout the study. The initial classification on day 22 will be cross-validated with the self-report stage of change measure, self-reported physical activity level from the International Physical Activity Questionnaire, and physical activity intention item from the baseline questionnaire

### Data and Compliance Monitoring

During EMA measurement bursts, participants are shown their compliance (ie, number of prompted surveys and number of completed surveys) in real time via the persistent study notification. Study staff perform real-time remote monitoring of participant compliance of all the above data collection modes. On a weekly basis, staff review data uploaded to the study server and contact participants by email or text message in the case of missing data to encourage compliance and address technical issues. The smartphone app is aware of the status of the phone and watch (ie, if the watch is being worn and sending data, if survey responses are being received, if devices are being properly charged daily), and the smartphone automatically prompts participants via notifications to encourage proper watch use throughout the study. Study staff withdraw participants from the study due to technical or participant issues that lead to poor data integrity, missing data, or ongoing low compliance. To aid attrition, after completing 9 months in the study, participants were allowed more leniency in missing smartphone surveys. Participants are sent a birthday card and quarterly newsletters to keep them engaged in the study to maintain compliance with study procedures. Participants are given a number and instructed to text study staff with any questions, concerns, or technical issues.

Given that this is one of the first studies to collect intensive longitudinal data over the course of an entire 12-month period, with intensive (ie, hourly) within-day self-reported measurement, we made the intentional choice to prioritize representation of the subject instead of representation of the population. Our goal was to minimize noncompliance and missing data, as both lead to an inaccurate representation of an individual’s daily life. Therefore, we decided to proactively remove participants from the study with low compliance given that this leads to biased data at the individual level. When recruiting for the study, we chose recruitment methods that would ensure a diverse sample, but we did not intend our sample to be representative of the entire population (given our intentional focus on those who could be compliant with the protocol). We acknowledge that our findings will not generalize to a broader population of young adults. However, our decision helps ensure that data will be generalizable to each individual and provide a reasonably accurate depiction of each individual’s daily life across a 12-month period. This study is part of early phase work that aims to examine the feasibility of the intensive data collection methods.

### Incentives

Participants can receive up to US $1260 for compliance with the study procedures. Each month (4-week period), participants can earn up to US $100, which includes US $20 for wearing the smartwatch at least 22 hours/day on at least 24 days and US $20 for answering at least 24 of the EOD EMA prompts. Participants receive US $10 for each EMA burst period they complete at least 8 prompts per day (2 EMA bursts per month; up to US $20). In addition, if a participant answers more than 11 EMA burst prompts on a given day, the participant receives a US $5 bonus per day (8 days per month; up to US $40/month). Participants are provided with their compensation electronically monthly. Participants who complete the 12-month data collection period may also keep their smartwatch at the end of the study. 

### Analytic Approach

#### Overview

Data analyses will combine idiographic (person-specific, data-driven) and nomothetic (generalizable, theory-driven) approaches.

#### Idiographic Approach

To test idiographic effects, statistical machine learning (ML) models will identify specific combinations of reflective and reactive variables predicting behavior for each person. A reduced set of key variables (included in the ML algorithms demonstrating ≥80% accuracy for at least half of the sample) will be selected for continuation into nomothetic testing, given that multilevel statistical models can only handle a limited number of variables. ML will also identify frequently occurring reflective-reactive variable pairings to be tested in targeted multilevel statistical interactions in the nomothetic phase.

#### Nomothetic Approach

We will use generalized linear mixed models (GLMMs) for nonnormal dependent variables, which adjust for clustering within subjects, allow for varying measurement schedules, and incorporate random effects. We will generate between-subject and within-subject versions of the time-varying predictors and moderators, representing their deviations from the subject and grand mean, respectively. To examine the likelihood of a *behavior episode*, we will test a 3-level model (level 1, occasion; level 2, measurement burst; level 3, person), and to examine the likelihood of a *behavior lapse*, we will test a 2-level version of this model (level 1, occasion; level 2, person). To examine whether the phase of behavior change (adoption vs maintenance) moderates within-subject effects, we will add within-subject and between-subject product interaction terms for the following time-varying moderators (ie, coded at the day level): being in the adoption versus maintenance phase (binary) and duration of maintenance (continuous).

#### Sample Size Estimation

ML algorithm training and testing are most effective when benchmark data sets fully represent the complexity of the phenomena being modeled. Given the exploratory nature of the person-specific ILD modeling, we aim to collect as much data as possible from each individual for the longest time frame reasonable given what we believe, based on prior work [55-57], is an acceptable EMA burden. We will have continuous data on reactive factors and behavior for 12 months, and for the EMA data on reflective factors, we will have up to 1901 observations per person (n=365 EOD EMA prompts+16×96 burst EMA prompts).

For the multilevel modeling, our most stringent sample size requirements will be to test between-subject effects. In G*Power (ver. 3.1.9.2) software, a sample size of 210 people (after accounting for 30% attrition) will have statistical power >0.80 with a 5% type I error rate to detect small effect sizes (odds ratios of 1.55-1.66) in two-sided logistic regressions. Given that the hypothesized within-subject effects will have much larger level-1 sample sizes (equivalent to the number of observations nested within people), we should have sufficient power to detect small to very small effects for all the remaining associations.

## Results

The study recruited young adults nationally (N=246). Rolling recruitment began in March 2020 and ended August 2021. Data collection will continue until August 2022.

Of the 332 participants who consented into the study, 51.5% (n=171) self-identified as a woman, 44.3% (n=147) as a man, and 4.2% (n=14) as nonbinary. Approximately half of the participants identified as nonwhite (n=184, 55.4%) and 26.0% (n=86) of participants identified as Hispanic or Latino. The mean age of participants was 23.6 (SD 3.2) years. Of the 290 participants who completed the baseline survey, 55.5% (n=161) were employed for wages, 49.3% (n=143) were students, and 13.5% (n=42) were out of work. Most participants lived at home with parents or guardians (135/290, 46.6%) or with their spouse or romantic partner (71/290, 24.5%). When describing their personal financial situation, 21.2% (61/290) of participants indicated that they “just meet” or “don’t meet” basic expenses.

Of the 246 participants fully enrolled in the study, 218 (88.6%) completed at least 3 months of data collection, 182 (74.0%) completed at least 6 months of data collection, and 148 (60.2%) completed at least 9 months of data collection to date. We expect that at least 50% of participants will complete the full 12 months of the study period.

## Discussion

The TIME study will be one of the first studies to use wearable smartphone and smartwatch technology to collect continuous data on physical activity, sedentary behavior, sleep, and their determinants across a 12-month period. The study aims to predict within-subject variation in the likelihood of behavior “episodes” and “lapses” as a function of reflective and reactive factors. We hypothesize that compared to models including only reflective variables, models that also include reactive variables will more accurately predict physical activity, sedentary, and sleep behavior episodes and lapses. Furthermore, we hypothesize that reflective variables will be less predictive (and reactive variables will be more predictive) of behavior episodes and lapses during the maintenance (vs adoption) phase of behavior change. Finally, we hypothesize that individuals who exhibit greater influence of reactive (versus reflective) variables on within-subject variation in behavior will be more likely to maintain the behavior across 12 months (without a relapse). This study advances beyond existing multimodal data sets owing to its intensive, innovative design. Out study is designed to test a dual-process model using a multimeasurement burst design across 12 months and passive data collection of physical activity, sedentary behavior, and sleep across the 24-hour activity cycle. Other existing multimodal studies were either of shorter duration, collected fewer EMA data points per day and per year, and/or had a smaller sample size.

Use of mobile technology to gather data with greater specification across time, situations, behaviors, and people has the potential to lead to the development of new theories and models that better explain health behavior than common frameworks. A new framework could provide opportunities to engage with questions about temporal specificity, including whether explanatory factors and behavior are temporally synchronous (ie, co-occur), the time scales across which effects unfold (eg, minutes/hours), the directionality of the effects (ie, antecedents vs consequences), and whether there are differences in the strength of effects across time (ie, time-varying effects). This framework may also address situational specificity such as determining under what combinations of conditions, contexts, or exposures (eg, environmental, affective, biological) the explanatory factors have the greatest effects (ie, time-varying moderators). The framework might further address behavioral specificity such as identifying the factors that are more predictive for different types of behavior (eg, leisure vs travel physical activity, homework vs watching TV) and person specificity, including identifying the sets of factors that are more predictive for certain people. Specification in these domains could offer a dramatic shift in the way that theories are developed, and would follow recent calls from the National Institutes of Health for more personalized/precision approaches to medicine [58,59].

Additionally, this study may help researchers understand the methodological and computational requirements of intensively adaptive interventions [60] or just-in-time adaptive interventions [61,62], which aim to deliver personalized behavior change strategies under the conditions when they will be most effective. The TIME study could yield information about the (1) number and composition of variables, (2) duration of the observation period, and (3) time delay between antecedent and behavior to accurately predict behavior. The study might also provide insight into whether there are discernible patterns or commonalities among people in the sets of explanatory factors of individual behaviors. The ability to reliably put people in these larger “bins,” if warranted, can (1) allow researchers to focus on developing a smaller number of intervention strategies targeting groups of people instead of separate interventions for each person, which can conserve resources and allow for greater efficiency; and (2) justify the foregoing of large and costly observation periods to determine unique sets of predictive factors for each individual prior to intervention development. Development of these targeted invention strategies is one of the many possible applications of ILD studies.

There are limitations of the study that must be acknowledged. Our use of GLMM and ML to perform data-driven analyses through running multiple models could be subject to overfitting and overgeneralization. Thus, the findings from this study should be interpreted with caution until they can be replicated in other studies. Additionally, our ML models will be limited by the diversity of the sample we have recruited and our results will lack generalizability to certain populations.

Overall, we anticipate that results from the TIME study will challenge current assumptions about, and yield new insights into, the fundamental structure and function of variables comprising health behavior theories, and eventually result in the development of more predictive models and personalized interventions targeting a wide variety of health behaviors.

## Appendix

Multimedia Appendix 1 Ecological momentary assessment questions presented in the TIME study.

## Abbreviations

API: application programming interface
AUC: area under the curve
CS: context-sensitive
EMA: ecological momentary assessment
EOD: end of day
GLMM: generalized linear mixed model
ILD: intensive longitudinal data collection
MIMS: Motion Independent Movement Summary
ML: machine learning
MVPA: moderate-to-vigorous physical activity
μEMA: microinteraction ecological momentary assessment
REDCap: Research Electronic Data Capture
SWaN: Sleep Wake and Non-wear algorithm
TIME: Temporal Influences on Movements and Exercise
USC: University of Southern California
