# Supplementary material for: Investigating Microtemporal Processes Underlying Health Behavior Adoption and Maintenance: Protocol for an Intensive Longitudinal Observational Study
Source: JMIR Res Protoc. 2022 Jul 14;11(7):e36666. doi: 10.2196/36666 (PMC9335174; doi:10.2196/36666)
Supplement: Multimedia Appendix 1 [file resprot_v11i7e36666_app1.docx]

| ***Variable*** | ***Item*** | ***Response Options*** | ***Prompt Type(s) and Frequency*** |
| --- | --- | --- | --- |
| **Global Questions** | | | |
| Affective and Feeling States | Right now, how **SAD** do you feel?  Over the past day, how **SAD** did you feel? | 1 = Not at all  2 = A little  3 = Moderately  4 = Quite a bit  5 = Extremely | Burst (100%) and End of Day (100%) |
|  | Right now, how **HAPPY** do you feel?  Over the past day, how **HAPPY** did you feel? |  | Burst (100%) and End of Day (100%) |
|  | Right now, how **FATIGUED** do you feel?  Over the past day, how **FATIGUED** did you feel? |  | Burst (100%) and End of Day (100%) |
|  | Right now, how **ENERGETIC** do you feel?  Over the past day, how **ENERGETIC** did you feel? |  | Burst (100%) and End of Day (100%) |
|  | Right now, how **RELAXED** do you feel?  Over the past day, how **RELAXED** did you feel? |  | Burst (100%) and End of Day (100%) |
|  | Right now, how **TENSE** do you feel?  Over the past day, how **TENSE** did you feel? |  | Burst (100%) and End of Day (100%) |
| Feeling Stress | Right now, how STRESSED do you feel?  Over the past day, how **STRESSED** did you feel? |  | Burst (100%) and End of Day (100%) |
|  | Right now, how **FRUSTRATED** do you feel?  Over the past day, how **FRUSTRATED** did you feel? |  | Burst (100%) and End of Day (100%) |
|  | Right now, how **NERVOUS** do you feel?  Over the past day, how **NERVOUS** did you feel? |  | Burst (100%) and End of Day (100%) |
| Attention | Right now, I feel **FOCUSED.**  Over the past day, I felt **FOCUSED.** |  | Burst (100%) and End of Day (100%) |
| Self-control | Right now, I feel **IN CONTROL.**  Over the past day, I felt like I could **RESIST** doing things that aren’t good for me. | 1 = Not at all  2 = A little  3 = Moderately  4 = Quite a bit  5 = Very much so | Burst (100%) and End of Day (100%) |
| Productivity | Right now, I am **PROCRASTINATING.**  Over the past day, I **PROCRASTINATED.** | 1 = Not at all  2 = A little  3 = Moderately  4 = Quite a bit  5 = Very much so | Burst (100%) and End of Day (100%) |
| Demands | Right now, I feel like I can’t get everything done.  Over the past day, I felt like I couldn’t get everything done. | 1 = Not at all  2 = A little  3 = Moderately  4 = Quite a bit  5 = Extremely | Burst (100%) and End of Day (100%) |
| Habit | Right now, I am following my usual routine.  Over the past day, I followed my usual routine. | 1 = Not at all  2 = A little  3 = Moderately  4 = Quite a bit  5 = Extremely | Burst (100%) and End of Day (100%) |
| Other behaviors | Over the past hour, I did the following things (choose all that apply) | 1= Used phone/tablet/computer  2= TV/videos/video games  3= Schoolwork  4= Work  5= Housework/chores  6= Exercise/physical activity  7= Read while sitting  8= Socialized (in person)  9= Socialized (phone call/video)  10=None of these things  11= Ate  12= Drank (non-alcoholic)  13= Drank alcohol  14= Slept/napped  15= Smoked/vaped  16= Rode in a car/vehicle  17= Did not do any of these things | Burst (100%) |
| Social Context | In the past hour, I was with (in person and/or virtual) | 1= friend(s)  2= romantic partner/spouse  3= classmate(s)/teacher(s)  4= coworker(s)  5= family member(s)/children  6= roommate(s)  7= neighbor(s)  8 = other type(s) of acquaintances  9=people I don’t know  10= alone | Burst (100%) |
| Illness | I was sick or ill today. | 1= Yes 2= No 3= Not sure | End of day (100%) |
| Miss work | I missed or took time off from work or school today. | 1= Yes 2= No 3= I didn’t have to go to work or school today | End of day (100%) |
| Travel | I traveled away from home today. | 1= Yes 2= No | End of day (100%) |
| Unusual circumstances | Over the past week, the following unusual events/circumstances have occurred (Select all that apply) | 1= Vacation 2= Travel 3= Illness/Injury of me 4= Illness/Injury of friend/family member 5=Increase in workload 6= Decrease in workload 7=Other major stressors 8= None of the above | End of Day (Sunday 100%) |
| Engagement | Answering surveys on my phone was disruptive today. | 0 = Not at all  1 = A little bit  2 = Somewhat  3 = Very much  4 = Extremely | Burst period end of day (25%) |
|  | This survey was disruptive. | 0 = Not at all  1 = A little bit  2 = Somewhat  3 = Very much  4 = Extremely | Burst (5%) |
| **Behavior-specific questions (each activity occurs as a set)  (Restricted to one behavior set per survey)** | | | |
| Affective Motivation  (Physical activity) | I **FEEL** like going on a walk or exercising in the next hour.  I **FEEL** like going on a walk or exercising tomorrow. | 1 = Not at all  2= A little  3= Quite a bit  4= Very much so  5= Already walking/exercising right now  1 = Not at all  2= A little  3= Quite a bit  4= Very much so | Burst PA set (33%)  End of day PA Set (25%) |
| Deciding/Deliberation (Physical activity) | I am going to take a walk or exercise in the next hour. | 1 = Yes  2= Trying to decide  3 = No  4 = Not thought about it  5= Already walking/exercising right now | Burst PA set (33%) |
| Intentions  (Physical activity) | I **INTEND** to do some walking or exercise tomorrow. | 1= Definitely will not  2= Probably will not  3= Might  4= Probably will  5= Definitely will | End of day PA Set (25%) |
| Plans (Physical Activity) | I have a **PLAN** for how I am going to do some walking or exercise tomorrow. | 1 = No  2= Not really  3= Sort of 4= Yes | End of Day PA Set (25%) |
| Goal- setting (Physical Activity) | I have a **GOAL** for how much walking or exercise I want to do this week. | 1 = No  2= Not really  3= Sort of  4= Yes | End of Day (Sunday only 100%) |
| Physical Activity Weekly Goal Subjective Report | Over the past week I have been able to achieve my walking/exercise goals**.** | 1= Yes 2= Maybe 3= No, but had a goal  4= No and I didn’t have a goal | End of Day (Sunday) |
| Physical Activity Daily Goal Subjective Report | I was able to achieve my walking/exercise goals today. | 1= Yes 2= Maybe 3= No, but had a goal  4= No and I didn’t have a goal | End of Day PA Set (25%) |
| Affective Motivation  (Sleep) | I **FEEL** like going to sleep in the next two hours. | 1 = Not at all  2= A little  3= Quite a bit  4= Very much so | End of day Sleep set (25%) |
| Deciding/Deliberation (Sleep) | I am going to go to sleep in the next two hours. | 1 = Yes  2= Trying to decide  3 = No  4 = Not thought about it | EOD Sleep set (25%) |
| Goal- setting (Sleep) | I have a goal for how much sleep I want to get this week. | 1 = No  2= Not really  3= Sort of  4= Yes | End of Day (Sunday only 100%) |
| Intentions  (Sleep) | I **INTEND** to get enough sleep tonight. | 1 = Definitely will not  2= Probably will not  3= Might  4= Probably will  5= Definitely will | EOD Sleep set (25%) |
| Sleep Goal Subjective Report | Over the past week I have been able to achieve my goal for how much sleep I want to get. | 1= Yes 2= Maybe 3= No, but had a goal  4= No and I didn’t have a goal | End of Day (Sunday only 100%) |
| Affective Motivation  (Sitting) | I **FEEL** like taking a break from sitting in the next hour.  I **FEEL** like sitting less tomorrow. | 1 = Not at all  2= A little  3= Quite a bit  4= Very much so  5= I’m not sitting right now  1 = Not at all  2= A little  3= Quite a bit  4= Very much so | Burst sitting set (33%)  End of day Sitting set (25%) |
| Deciding/Deliberation  (Sitting) | I am going to take a break from sitting in the next hour. | 1 = Yes  2= Trying to decide  3 = No  4 = Not thought about it  5= I’m not sitting right now | Burst sitting set (33%) |
| Intentions  (Sitting) | I **INTEND** to limit my sitting tomorrow. | 1=Definitely will not  2= Probably will not  3= Might  4= Probably will  5= Definitely will | End of day sitting set (25%) |
| Goal- setting (Sitting) | I have a **GOAL** for how much I will limit my sitting this week. | 1 = No  2= Not really  3= Sort of  4= Yes | End of Day (Sunday only 100%) |
| Plans (Sitting) | I have a **PLAN** for how I will sit less tomorrow. | 1 = No  2= Not really  3= Sort of  4= Yes | End of day sitting set (25%) |
| Affective Motivation  (Healthy eating) | I **FEEL** like eating healthy foods at my next meal.  I **FEEL** like eating healthy tomorrow. | 1 = Not at all  2= A little  3= Quite a bit  4= Very much so | Burst (33%)  End of day Eating set (25%) |
| Intentions  (Healthy eating) | I **INTEND** to eat healthy foods tomorrow. | 1= Definitely will not  2= Probably will not  3= Might  4= Probably will  5= Definitely will | End of day Eating set (25%) |
| Deciding/Deliberation (Healthy eating) | I am going to eat healthy foods at my next meal. | 1 = Yes  2= Trying to decide  3 = No  4 = Not thought about it | Burst (33%) |
| Goal- setting  (Healthy eating) | I have a **GOAL** for what type of healthy foods I want to eat this week. | 1 = No  2= Not really  3= Sort of  4= Yes | End of Day (Sunday only 100%) |
| Plans  (Healthy eating) | I have a **PLAN** for how I am going to eat healthy foods tomorrow. | 1 = No  2= Not really  3= Sort of  4= Yes | End of Day Eating set (25%) |
| Adoption/Maintained (Physical Activity) | Over the past week which of the following BEST describes your behavior with respect to your physical activity habits: | 1= I was working on developing a better physical activity routine  2= I was maintaining an existing physical activity routine  3= Neither of these  OR  I didn't really have a physical activity routine | End of Day (Sunday 100%) |
| Adoption/Maintained (Sitting) | Over the past week which of the following BEST describes your behavior with respect to your sitting habits: | 1= I was working on developing a better routine of sitting less each day  2= I was maintaining an existing routine of limited sitting each day  3- Neither of these  OR  I didn't really have a routine regarding my amount of sitting each day | End of Day (Sunday 100%) |
| Adoption/Maintained (Sleep) | Over the past week which of the following BEST describes your behavior with respect to your sleep habits: | 1= I was working on developing better a sleep routine  2= I was maintaining an existing sleep routine  3= Neither of these  OR  I didn't really have a sleep routine | End of Day (Sunday 100%) |
| Adoption/Maintained (Healthy eating) | Over the past week which of the following BEST describes your behavior with respect to your eating habits: | 1= I was working on developing a better or healthier way of eating  2= I was maintaining an existing way of eating  3= Neither of these  OR  I didn't really have a regular way of eating diet | End of Day (Sunday 100%) |

**COVID Questions (Every Sunday EOD 100%)**

1. **Over the past week, how much were you lonely?**

Not at all, A little, Moderately, Quite a bit, Extremely

1. **Over the past week, to what extent has the COVID-19 situation affected your physical health?**

Not at all, A little, Moderately, Quite a bit, Extremely

1. **Over the past week, which of the following are true about your physical health? (Viral symptoms typically include fever, body aches, cough, fatigue, chills, digestive issues)**
   1. I am not experiencing viral symptoms
   2. I am currently experiencing mild-moderate viral symptoms
   3. I am currently experiencing severe viral symptoms
2. **Over the past week, which of the following behaviors have you been performing to prevent the spread of COVID-19? (check all that apply)**
   1. Avoiding indoor social gatherings with anyone who does not live with me
   2. Avoiding outdoor social gatherings with anyone who does not live with me
   3. Avoiding any places with 10 or more people
   4. Staying at least 6 feet away from other people who do not live with me
   5. Avoiding physical contact with other people who do not live with me
   6. None of the above
3. **Over the past week, which of the following did you do because of tahe COVID-19 (coronavirus) outbreak? (check all that apply)**
   1. Avoided outdoor spaces (e.g., parks, beaches, forests)
   2. Avoided public spaces (e.g., museums, playgrounds, theatres)
   3. Avoided restaurants, coffee shops, bars
   4. Avoided gyms, fitness centers, yoga studios
   5. Avoided participating in team sports
   6. None of the above
4. **Over the past week, what else of the following did you do because of the COVID-19 (coronavirus) outbreak? (check all that apply)**
   1. Avoided going for walks or other outdoor exercise activity
   2. Avoided grocery stores, pharmacies
   3. Avoided public transportation
   4. Avoided indoor exercise classes or recreational sports
   5. Avoided in-person events in the community
   6. Avoided in-person religious services
   7. None of the above
5. **Over the past week, which of the following behaviors have you been performing? (check all that apply)?**
   1. Wearing masks or face covering when around those who live with me
   2. Wearing masks or face covering when around those who do not live with me
   3. Washing hands with soap and water whenever I return home
   4. Used hand sanitizer
   5. Coughed/sneezed into your elbow
   6. Avoiding non-ventilated spaces
   7. None of the above
6. **Over the past week, where did you do physical activity/exercise (choose all that apply)?**
   1. Inside my home or garage
   2. In my yard or driveway
   3. On the sidewalks and roads IN my neighborhood
   4. On the sidewalks and roads OUTSIDE my neighborhood
   5. At a gym or fitness center
   6. At a park, trail, or beach
   7. At an outdoor sports facility
   8. Other places
   9. None of the above
7. **Over the past week, with whom did you do physical activity/exercise in the same physical space (choose all that apply)?**
   1. Spouse/partner
   2. Child(ren)
   3. Other family members (e.g., cousins, siblings, aunts, uncles)
   4. Friends
   5. Coworkers
   6. Other people
   7. None of the above
8. **Over the past week, did you use any remote/streaming services to facilitate your physical activity/exercise? (check all that apply)**
   1. Yes – with friends or family
   2. Yes –from my gym/fitness facility
   3. Yes – I streamed online workout classes
   4. Yes – I played a game online that involves physical activity
   5. No – I did not use any remote/streaming services
9. **Over the past week, on how many days did you use any remote/streaming services to facilitate your physical activity/exercise?**
   1. 0 days
   2. 1 day
   3. 2 days
   4. 3 days
   5. 4 days
   6. 5 or more days
10. **Over the past week, how many days did you spend walking a dog?**
    1. 0 days
    2. 1 day
    3. 2 days
    4. 3 days
    5. 4 days
    6. 5 or more days
11. **Over the past week, the COVID-19 outbreak has affected how much PHYSICAL ACTIVITY/EXERCISE I got compared to before the COVID-19 outbreak. I exercised…**
    A lot more, more, neither more nor less, less, a lot less
12. **Over the past week, the COVID-19 outbreak affected how much SITTING I did compared to before the COVID-19 outbreak. I sat…**A lot more, more, neither more nor less, less, a lot less
13. **Over the past week, the COVID-19 outbreak has affected how much SLEEP I get compared to before the COVID-19 outbreak. I slept…**A lot more, more, neither more nor less, less, a lot less
14. **Over the past week, what has been the overall level of impact to your daily life due to the COVID-19 outbreak?**Not at all, Very little, Moderate, Quite a bit, Extremely
15. **Over the past week, what has been your overall level of stress related to the COVID-19 outbreak?**Not at all, Very little, Moderate, Quite a bit, Extremely
